# De Novo Human Angiotensin-Converting Enzyme 2 Decoy NL-CVX1 Protects Mice From Severe Disease After Severe Acute Respiratory Syndrome Coronavirus 2 Infection

**DOI:** 10.1093/infdis/jiad135

**Published:** 2023-06-05

**Authors:** Maria Rebelo, Cong Tang, Ana R Coelho, Carlos Labão-Almeida, Matthias M Schneider, Laurie Tatalick, Pedro Ruivo, Marta Pires de Miranda, Andreia Gomes, Tânia Carvalho, Matthew J Walker, Hannes Ausserwoeger, J Pedro Simas, Marc Veldhoen, Tuomas P J Knowles, Daniel-Adriano Silva, David Shoultz, Gonçalo J L Bernardes

**Affiliations:** Instituto de Medicina Molecular João Lobo Antunes, Faculdade de Medicina, Universidade de Lisboa, Lisboa, Portugal; Instituto de Medicina Molecular João Lobo Antunes, Faculdade de Medicina, Universidade de Lisboa, Lisboa, Portugal; Instituto de Medicina Molecular João Lobo Antunes, Faculdade de Medicina, Universidade de Lisboa, Lisboa, Portugal; Instituto de Medicina Molecular João Lobo Antunes, Faculdade de Medicina, Universidade de Lisboa, Lisboa, Portugal; Yusuf Hamied Department of Chemistry, University of Cambridge, Cambridge, United Kingdom; Laurie Tatalick Consulting, Redmond, Washington, USA; Instituto de Medicina Molecular João Lobo Antunes, Faculdade de Medicina, Universidade de Lisboa, Lisboa, Portugal; Instituto de Medicina Molecular João Lobo Antunes, Faculdade de Medicina, Universidade de Lisboa, Lisboa, Portugal; Instituto de Medicina Molecular João Lobo Antunes, Faculdade de Medicina, Universidade de Lisboa, Lisboa, Portugal; Histopathology Unit, Champalimaud Research, Lisboa, Portugal; Neoleukin, Seattle, Washington, USA; Yusuf Hamied Department of Chemistry, University of Cambridge, Cambridge, United Kingdom; Instituto de Medicina Molecular João Lobo Antunes, Faculdade de Medicina, Universidade de Lisboa, Lisboa, Portugal; Católica Biomedical Research and Católica Medical School, Universidade Católica Portuguesa, Lisboa, Portugal; Instituto de Medicina Molecular João Lobo Antunes, Faculdade de Medicina, Universidade de Lisboa, Lisboa, Portugal; Yusuf Hamied Department of Chemistry, University of Cambridge, Cambridge, United Kingdom; Neoleukin, Seattle, Washington, USA; Neoleukin, Seattle, Washington, USA; Instituto de Medicina Molecular João Lobo Antunes, Faculdade de Medicina, Universidade de Lisboa, Lisboa, Portugal; Yusuf Hamied Department of Chemistry, University of Cambridge, Cambridge, United Kingdom

**Keywords:** COVID-19, SARS-CoV-2, de novo protein decoys, k18-hACE2 mice, treatment

## Abstract

The emergence of novel variants of severe acute respiratory syndrome coronavirus 2 (SARS-CoV-2) underscores the need to investigate alternative approaches to prevent infection and treat patients with coronavirus disease 2019. Here, we report the preclinical efficacy of NL-CVX1, a de novo decoy that blocks virus entry into cells by binding with nanomolar affinity and high specificity to the receptor-binding domain of the SARS-CoV-2 spike protein. Using a transgenic mouse model of SARS-CoV-2 infection, we showed that a single prophylactic intranasal dose of NL-CVX1 conferred complete protection from severe disease following SARS-CoV-2 infection. Multiple therapeutic administrations of NL-CVX1 also protected mice from succumbing to infection. Finally, we showed that infected mice treated with NL-CVX1 developed both anti-SARS-CoV-2 antibodies and memory T cells and were protected against reinfection a month after treatment. Overall, these observations suggest NL-CVX1 is a promising therapeutic candidate for preventing and treating severe SARS-CoV-2 infections.

## Teaser

We show that NL-CVX1, a de novo human angiotensin-converting enzyme 2 protein decoy that blocks viral cell invasion, prevents severe disease after severe acute respiratory syndrome coronavirus 2 (SARS-CoV-2) infection in mice and may constitute a potent anti–SARS-CoV-2 treatment.

The emergence of coronavirus disease 2019 (COVID-19) constitutes a major global public health crisis. Unprecedented global efforts have led to rapid development of effective vaccines for COVID-19 [1], which have reduced rates of infection, disease severity, hospitalization, and death [2]. However, novel variants of severe acute respiratory syndrome coronavirus 2 (SARS-CoV-2), continue to emerge, compromising efforts made so far [3–5]. The development of novel targeted therapeutics to fight COVID-19 is of utmost importance.

SARS-CoV-2 cell entry relies on the interaction of its spike protein with angiotensin-converting enzyme 2 (ACE2) receptors on the cell surface [6, 7]. A novel de novo protein design strategy was used to develop a human ACE2 (hACE2) decoy to neutralize SARS-CoV-2 [8]. The decoy, originally referred to as CTC-445.2d and here as NL-CVX1, was shown to bind to the receptor-binding domain (RBD) of the spike protein with nanomolar affinity and high specificity, preventing SARS-CoV-2 entry into cells [8]. Furthermore, NL-CVX1 was designed to be intrinsically resilient to viral mutation escape because the decoy replicates the spike protein interface in hACE2 and thus may have antiviral activity across diverse SARS-CoV-2 variants.

In the current study, we evaluated the preclinical efficacy of NL-CVX1 using an hACE2 transgenic mouse model of SARS-CoV-2 infection (K18-hACE2) [9]. Prophylactic intranasal administration of NL-CVX1 conferred complete protection from severe disease after infection with both ancestral SARS-CoV-2 and the Delta variant of concern (VOC). Repeated therapeutic administration of NL-CVX1 also protected mice from body weight loss and infection-related death. Furthermore, we showed that a single prophylactic NL-CVX1 administration protected mice from reinfection a month later. Overall, these observations indicate that NL-CVX1 may constitute a novel approach to prevent SARS-CoV-2 infection and control COVID-19.

## MATERIAL AND METHODS

### Cells and Viruses

Vero CCL-81 cells (provided by J. P. S.) were maintained in Dulbecco's modified Eagle medium supplemented with 10% fetal bovine serum, 1% penicillin-streptomycin, and 1% glutamax at 37°C and 5% carbon dioxide. The ancestral SARS-CoV-2 was isolated from a Portuguese patient (internal reference 606_IMM ID_5452). The Alpha variant (NR-54000; lineage B.1.1.7) was contributed by Bassam Hallis, and the Delta (NR-55611; lineage B.1.617.2) variant by Richard Webby, PhD and Anami Patel, PhD, obtained through BEI Resources (National Institute of Allergy and Infectious Diseases, National Institutes of Health). The Omicron variant (lineage B.1.1.529, sublineage BA.1) was obtained through a World Health Organization BioHub Facility: Spiez Laboratory in Switzerland. Infectious stocks were expanded in Vero CCL-81 cells. Work with infectious SARS-CoV-2 was conducted in a biosafety level 3 laboratory following European Union (EU) legislation (directives 2000/54/EC and EU 2020/739) and World Health Organization guidelines.

### K18-hACE2 Mouse Model of SARS-CoV-2 Infection

We used K18-hACE2 (strain B6.Cg-Tg(K18-ACE2)2Prlmn/J) mice (from Charles River, to investigate the efficacy of the hACE2 decoy NL-CVX1 against SARS-CoV-2 infection in the presence of the human version of hACE2 protein. Animal studies were conducted in compliance with the EU and national legislation and were approved by the Portuguese veterinary authority (license no. 01878/2021) and the institutional animal ethics committee. Male and female K18-hACE2 mice, 7–11 weeks old, were used. Mice reaching 80% of initial body weight or severe signs of illness (eg, back arching, shaking, ruffling of fur, and/or reduced mobility) were considered to have reached humane end points and were euthanized.

### NL-CVX1 Preparation and Administration to K18-hACE2 mice

NL-CVX1 stock was synthesized as described elsewhere [8] and stored at −80°C. NL-CVX1 stocks were thawed by immersion at 37°C for 1 hour and then measured at 280 nm, using an absorbance coefficient of 0.314 and diluted in phosphate-buffered saline with 0.01% Tween 80 (Sigma) to achieve 0.5, 2, and 5 mg/mL. NL-CVX1 solution was stored at 4°C and used within 7 days. At defined time points, mice were anesthetized with isoflurane, and 50 μL of NL-CVX1 was administered intranasally.

### Prophylactic Exposure Studies With Ancestral SARS-CoV-2 and Delta and Omicron VOCs

For the dose-response study, on day 0 mice were dosed with 50 µL of vehicle (phosphate-buffered saline plus 0.01% Tween 80) or NL-CVX1 at 25, 100, or 250 µg, 1 hour before intranasal inoculation with 10^4^ plaque-forming units (PFUs) of ancestral SARS-CoV-2. The inoculum of approximately 1 × 10^4^ PFUs was chosen based on published literature [9]. NL-CVX1 concentrations were selected based on previous toxicology studies in rats (Daniel Adriano-Silva, PhD, November 2020, unpublished). Between days 5 and 6, when vehicle-treated mice reached humane end points, 3 mice from each NL-CVX1 treated groups were euthanized, and the other 3 were monitored until day 21. For the Delta VOC study, mice were inoculated with 0.5 × 10^4^ PFUs and administered vehicle (n = 3) or 250 µg of NL-CVX1 (n = 6). Three of 6 NL-CVX1–treated mice were euthanized on day 7, and the other 3 were monitored until day 21. For the Omicron VOC study, mice were inoculated with 1.5 × 10^3^ PFUs and administered vehicle (n = 12) or 250 µg of NL-CVX1 (n = 12). Six mice from each group were euthanized on day 3, and the other 6 on day 6. On euthanasia day, the left lung was collected and homogenized for viral load quantification (Supplementary Methods 1), and the right lung was harvested for histopathological analysis.

### Measurement of Lung Viral Load by Polymerase Chain Reaction

Lung viral load was quantified using nucleocapsid (N) and envelope (E) genes, normalized to 18S (primers sequence in Supplementary methods 2). RNA was extracted using a Viral RNA Isolation kit (catalogue no. MB40701; NZYTech), and complementary DNA (cDNA) was synthesized using First-Strand cDNA Synthesis kit (catalogue no. MB12502; NZYTech). Polymerase chain reaction (PCR) analysis was performed using the PowerUp SYBR Green Master Mix with the Applied Biosystems RT-PCR 7500Fast Thermocycler.

### Quantification of Lung Viral Load by Plaque Assay

Lung homogenates were 10-fold serially diluted (10^−1^ to 10^−6^) in 2.5% fetal bovine serum supplemented with Dulbecco's modified Eagle medium, and 0.5 mL of each dilution was incubated with 80% confluent Vero CCL-81 cells in 6-well plates, for 1 hour at 37°C. Viral inoculum was removed, and an overlay of 1.25% carboxymethylcellulose was added. Cells were incubated for 4 days at 37°C. After incubation, carboxymethylcellulose was removed and cells were fixed with 4% formaldehyde and stained with 0.1% toluidine blue.

### Histopathology

The right lung was fixed in 10% neutral buffered formalin, processed for paraffin embedding, sectioned at 4 µm, and stained with hematoxylin-eosin. Lesions were scored by a pathologist blinded to experimental groups, according to previously published criteria [10] (Supplementary Methods 3). Representative photomicrographs were obtained using NDP.view2 software (Hamamatsu) in slides digitally scanned with the NanoZoomerSQ scanner.

### Immune Characterization Study

On day 0, mice were dosed with NL-CVX1 at 250 µg (n = 10), 1 hour before SARS-CoV-2 inoculation. Vehicle-infected (n = 5) and noninfected (n = 5) controls groups were also included. Inflammatory cells were isolated from spleens and right lungs on days 6 and 31, and the relative abundance of myeloid and lymphoid cell populations was assessed by means of flow cytometry. Blood samples were collected by cardiac puncture, and serum samples obtained for cytokine profiling (day 6) and/or antibody quantification (days 10, 21, and 31).

### Reinfection Study

On day 0, mice were dosed with NL-CVX1 at 250 µg (n = 16), 1 hour before SARS-CoV-2 inoculation. On day 31, mice were reinfected with 10 PFUs (n = 3) or 1 × 10^4^ PFUs of ancestral SARS-CoV-2 (n = 5), 0.5 × 10^4^ PFUs of the Alpha VOC (n = 3), or 1 × 10^4^ PFUs of the Delta VOC (n = 8). Mice were monitored for 21 days.

### Therapeutic Exposure Study

On day 0, mice were inoculated with ancestral SARS-CoV-2 (n = 8) or the Delta VOC (n = 8). Two therapeutic dosing schemes were investigated in which mice were (1) were administered 1 daily dose of NL-CVX1 at 250 µg for 3 days (n = 3) or (2) dosed with NL-CVX1 at 250 µg every 12 hours after inoculation (n = 5), for 5 days. Mice were monitored until day 21.

### Staining of Inflammatory Cells for Flow Cytometry

On day 6 and 31, cells were isolated from the right lung and spleens of mice and incubated with an extracellular antibody mix for inflammatory cell staining (Supplementary Methods 4 and 5). For fixation, eBioscience IC Fixation/Permeabilization buffer was used for 30 minutes at room temperature (RT).

For intracellular staining, cells were permeabilized twice using permeabilization buffer at ×1 (Thermo Fisher Scientific) and incubated with anti-mouse Foxp3 antibody (clone FJK-16s), for 1 hour at RT. After incubation, cells were washed with permeabilization buffer, centrifuged at 620*g* at RT for 2 minutes, and resuspended in fluorescence-activated cell sorting buffer for flow cytometry analysis. Samples were analyzed with a BD LSRFortessa analyzer, and data analysis was performed using FlowJo version 10 software (BD Biosciences) (Supplementary Figure 2 and 3).

### Cytokine and Chemokine Measurement

Heat inactivated serum samples were shipped to Eve Technologies (Canada), and the Mouse Cytokine/Chemokine 31-Plex Discovery Assay was performed (biomarker list in Supplementary Methods 6).

### Enzyme-Linked Immunosorbent Assays or Quantification of Immunoglobulin G antibodies

Serum samples were analyzed for antibodies, using SARS-CoV-2 Spike protein followed by titer determination, as described elsewhere [11, 12] (Supplementary Methods 7). Anti-spike immunoglobulin G levels were quantified by 2-fold serial dilutions. Each plate contained positive and negative quality control samples, composed of a pool of positive and negative samples, respectively.

## RESULTS

### Prophylactic Activity of a Single Intranasal Dose of NL-CVX1 in Mice Infected with SARS-CoV-2

**Figure 1.**
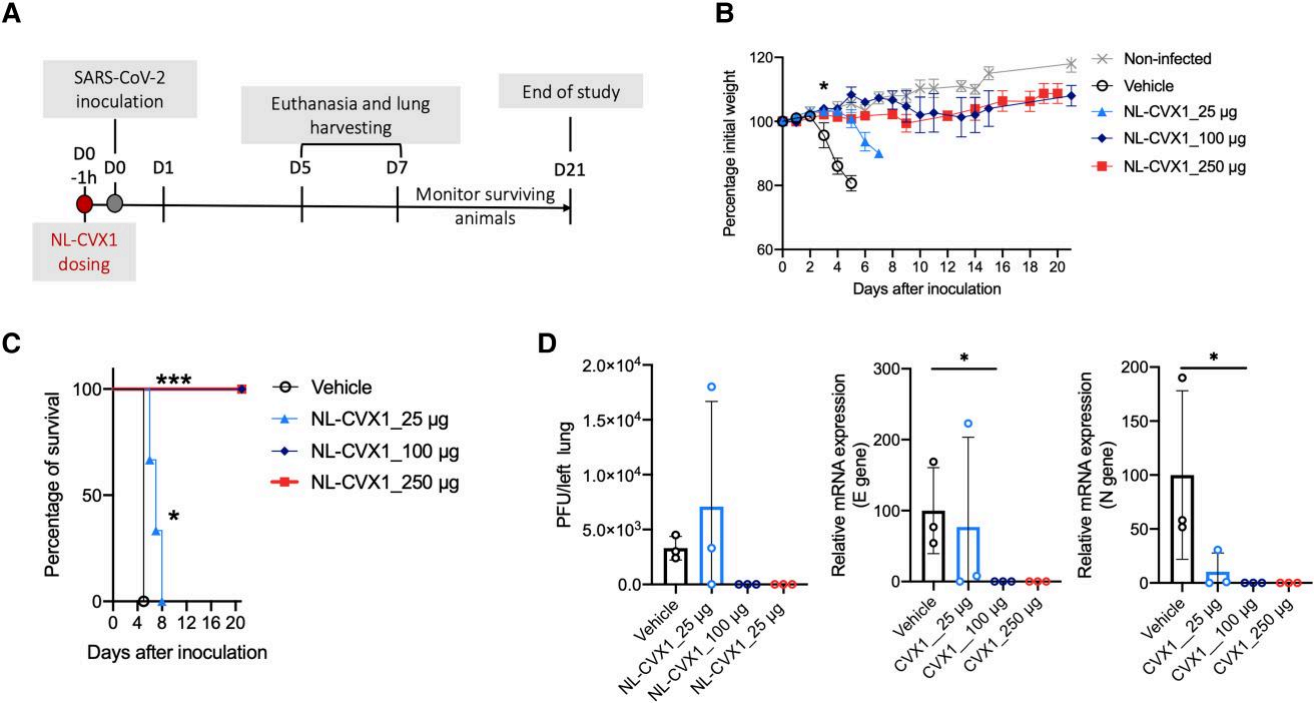
Single-dose intranasal prophylaxis with NL-CVX1 protects mice from lethal infection with ancestral severe acute respiratory syndrome coronavirus 2 (SARS-CoV-2). *A*, Female K18–human angiotensin-converting enzyme 2 (hACE2) mice, 7–8 weeks old, were intranasally administered NL-CVX1 or vehicle, 1 hour (1h) before intranasal inoculation with 10^4^ plaque-forming units (PFUs) of SARS-CoV-2. Mice were monitored for weight loss, disease, and death for 21 days. Abbreviations: d0, d1 (etc), day 0, day 1, (etc). *B*, Body weight change (mean with standard error of the mean) after administration of vehicle (*clear circle*; n = 3) or NL-CVX1 at 25 (*triangle*; n = 6), 100 (*diamond*; n = 6), or 250 μg (*square*; n = 6). *P < .05 (multiple *t* test). *C*, Percentage survival after vehicle or NL-CVX1 administration. *P < .05; ***P < .001 (Mantel-Cox test). *D*, Lung viral loads (mean with standard deviation) on day 5 after infection were determined by plaque assay and by polymerase chain reaction targeting the envelope (E) and nucleocapsid (N) genes. *P < .05 (Kruskal-Wallis test). Abbreviation: mRNA, messenger RNA.

**Figure 2.**
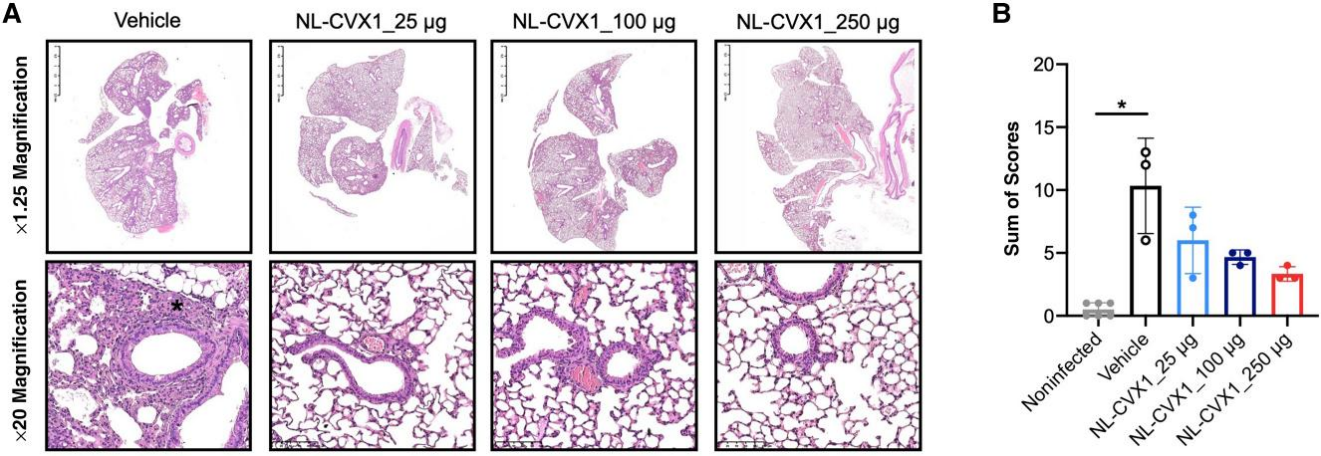
Histopathology of mice infected with ancestral severe acute respiratory syndrome coronavirus 2 (SARS-CoV-2) and administered a single intranasal dose of NL-CVX1. *A*, Subgross and high-magnification representative photomicrographs (3 mice per group) of lung 5 days after infection (hematoxylin-eosin stain). NL-CVX1 treatment resulted in less pulmonary edema, hyaline membrane formation, proliferation of bronchiolar epithelium, hemorrhage, and neutrophil infiltration. *B,* Sum of histopathological scores (mean with standard deviation) for lung injury and inflammation in noninfected K18–human angiotensin-converting enzyme 2 (hACE) mice and K18-hACE mice inoculated with SARS-CoV-2 and administered vehicle or NL-CVX1. *P < .05 (Kruskal-Wallis test followed by Dunn multiple comparison).

We investigated the prophylactic dose-response effect of a single administration of NL-CVX1 at 25, 100, or 250 μg at 1 hour before SARS-CoV-2 inoculation (Figure 1*A*). Infected mice administered vehicle showed important weight loss on day 3 and reached a humane end point at day 5 (Figure 1*B* and 1*C*). Infected mice administered with 25 μg of NL-CVX1 showed a statistically significant 2-day delay in body weight loss yet developed severe disease and were euthanized on day 6 and 7 (Figure 1*C*). Infected mice given 100 or 250 μg of NL-CVX1 did not lose any weight and showed a body weight curve similar to that in noninfected mice (Figure 1*B*). In agreement with in vivo observations, lung viral load quantification by plaque assay and PCR on day 5 showed that infectious virus or viral RNA was not detected in mice given 100 or 250 μg of NL-CVX1, contrasting with the vehicle and low-dose groups, in which higher levels of lung viral load were measured (Figure 1*D*). Histopathological analysis of lung at day 5 showed that a single NL-CVX1 administration reduced inflammation (Figure 2).

**Figure 3.**
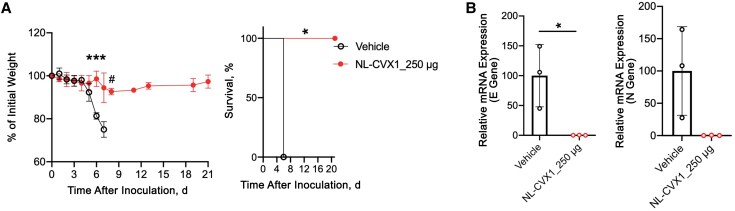
Single-dose intranasal prophylaxis of NL-CVX1 protects mice from lethal infection with the Delta variant of concern (VOC). *A*, Body weight change (mean with standard error of the mean) and survival after administration of vehicle (*clear circle*; n = 3) or NL-CVX1 at 250 μg (*filled circle*; n = 6) in mice infected with the Delta VOC. Body weight changes between experimental groups were analyzed statistically using multiple *t* tests; body weight changes in each group, compared with day 0, were analyzed using Kruskal-Wallis tests, followed by Dunn multiple comparison; and survival was analyzed using Mantel-Cox tests. *P < .05; ***P < .001; #*P* = .05. *B,* Lung viral loads (mean with standard deviation), determined by polymerase chain reaction (PCR) targeting the envelope (E) and nucleocapsid (N) genes on day 7 after infection. *P < .05 (unpaired *t* test). Abbreviation: mRNA, messenger RNA.

**Figure 4.**
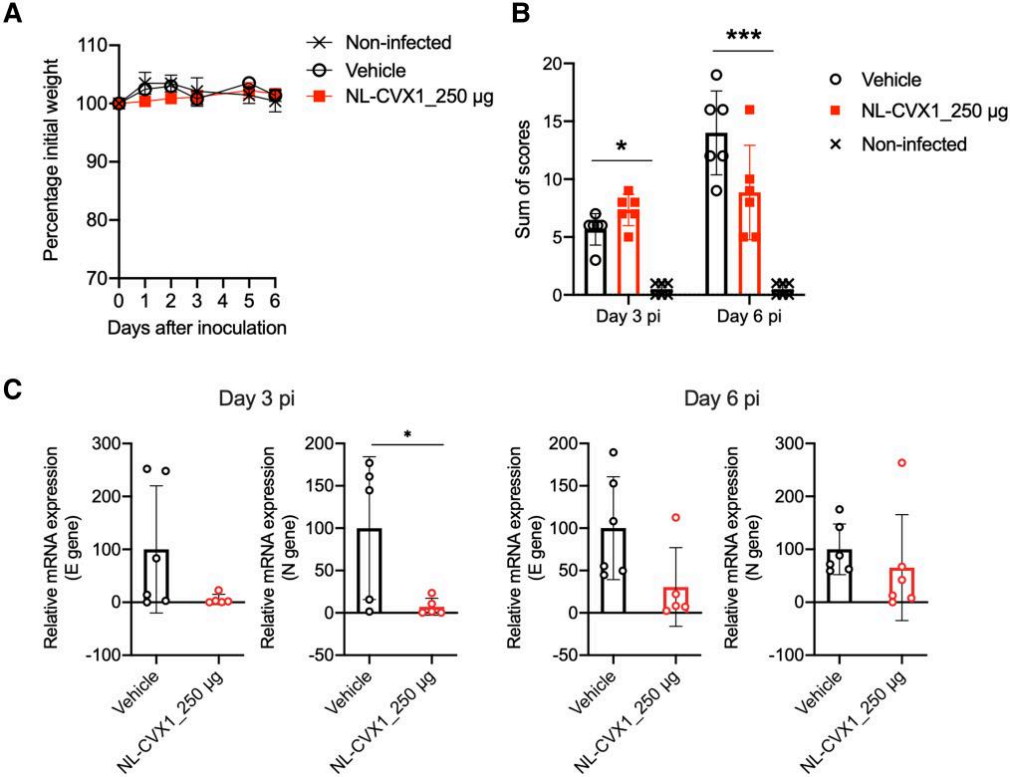
Single-dose intranasal prophylaxis of NL-CVX1 protects mice from lethal infection with the Omicron variant of concern (VOC). *A*, Body weight change (mean with standard error of the mean) after administration of vehicle (*clear circle*; n = 6) or NL-CVX1 at 250 μg (*filled circle*; n = 6) in mice infected with the Omicron VOC. *B*, Sum of histopathological scores (mean with standard deviation) for lung injury and inflammation of noninfected K18– human angiotensin-converting enzyme 2 (hACE) mice and K18-hACE mice inoculated with the Omicron VOC and administered vehicle or NL-CVX1 on days 3 and 6 after infection. *P < .05; ***P < .001 (Kruskal-Wallis test followed by Dunn multiple comparison). *C*, Lung viral loads (mean with standard deviation) determined by polymerase chain reaction targeting the envelope (E) and nucleocapsid (N) genes on days 3 and 6 after infection. *P < .05 (unpaired *t* test). Abbreviation: mRNA, messenger RNA.

We also investigated the efficacy of NL-CXV1 against the Delta and Omicron VOCs. A single prophylactic dose of NL-CVX1 at 250 μg prevented mice infected with the Delta VOC from exhibiting signs of disease and succumbing to infection (Figure 3*A*). Even though a significant decrease in body weight occurred on day 6, no further weight was lost until the end of the study. Moreover, low levels of viral RNA were detected in the lungs of NL-CXV1–treated mice infected with the Delta VOC (Figure 3*B*). As reported, we observed that Omicron infection in K18-hACE2 mice is milder and does not lead to body weight loss [13]. Therefore, no differences in body weight were observed between vehicle-treated and NL-CVX1–treated mice (Figure 4*A*). On day 6, histopathological analysis of lung showed and important reduction in lesion burden in NL-CVX1–treated mice (Figure 4*B*). However, unlike noninfected controls, NL-CVX1–treated animals displayed lung pathology, suggesting that a single dose of NL-CVX1 at 250 μg does not fully protect mice from infection. PCR analysis on day 3 showed 90% reduction in viral RNA in NL-CVX1–treated mice, while on day 6 differences between vehicle-treated and NL-CVX1–treated animals were smaller (Figure 4*C*).

### Characterisation of the Immune Response in Mice Infected with SARS-CoV-2 and Administered with NL-CVX1

**Figure 5.**
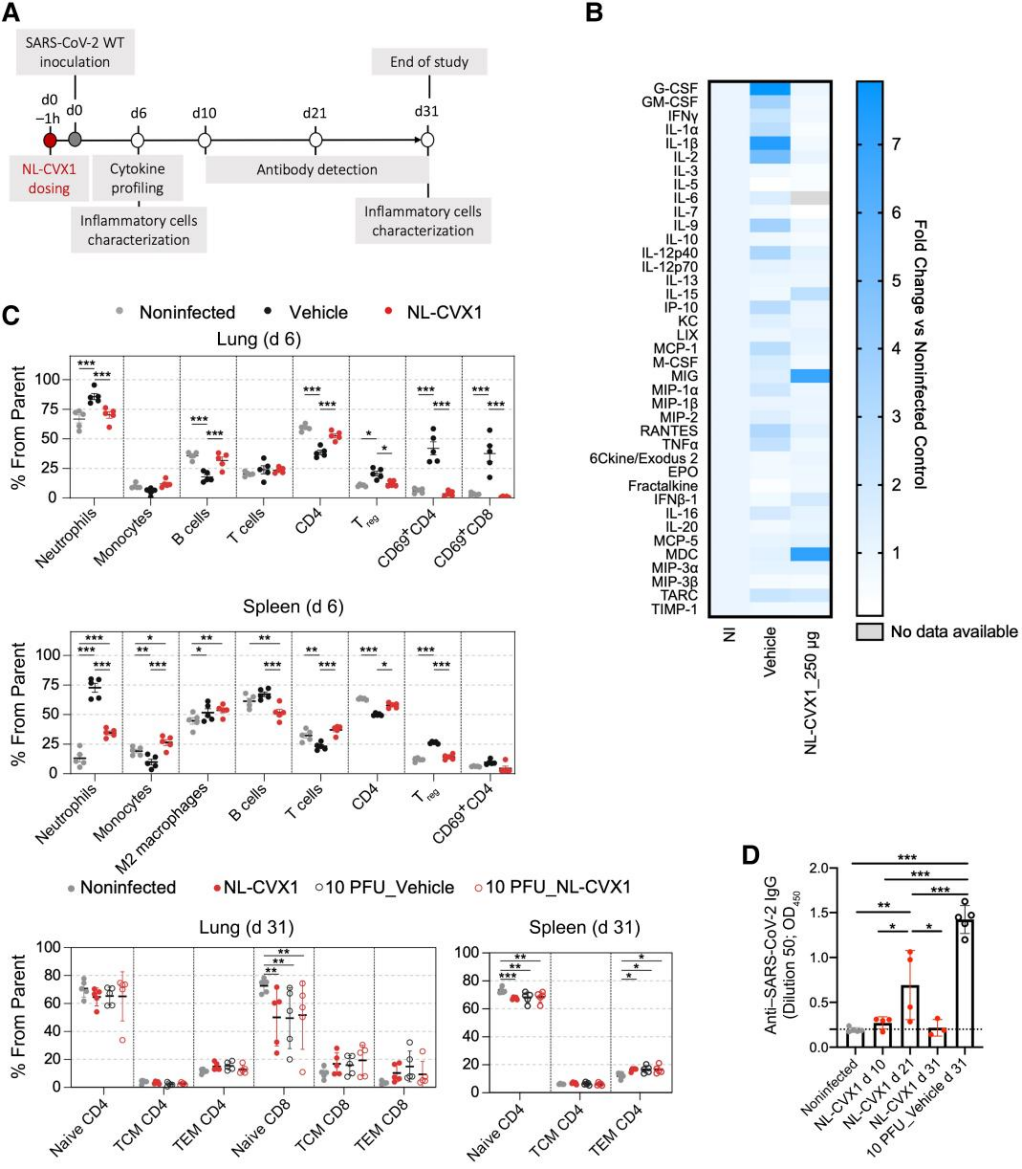
Immune characterization of NL-CVX1 treated mice. *A*, Female K18– human angiotensin-converting enzyme 2 (hACE2) mice, aged 7–11 weeks, were intranasally administered NL-CVX1 at 250 μg or vehicle 1 hour (1h) before intranasal inoculation with 10^4^ plaque-forming units (PFUs) or 10 PFUs of ancestral severe acute respiratory syndrome coronavirus 2 (SARS-CoV-2) (wild type [WT]). *B–D,* Serum samples were collected on day 6 for cytokine profiling (*B*), lung and spleen immune cells were isolated and analyzed by flow cytometry on days 6 and 31 (*C*), and antibodies against SARS-CoV-2 spike protein were measured on days 10, 21, and 31 (*D*). *B*, Fold change heat map of serum cytokine concentrations on day 6, measured by multiplex assay of SARS-CoV-2–infected mice treated with NL-CVX1 or vehicle. Fold changes were calculated from normalization to noninfected (NI) mice (5 mice per group). Abbreviations: G-CSF, Granulocyte colony-stimulating factor; GM-CSF, Granulocyte-macrophage colony-stimulating factor; IFNγ, Interferon gamma; IL, Interleukin; IP-10, Interferon gamma-induced protein 10; KC, keratinocyte-derived chemokine; LIX, lipopolysaccharide-induced CXC chemokine; MCP, Monocyte chemoattractant proteins; M-CSF, Macrophage colony-stimulating factor; MIG, monokine induced by gamma interferon; MIP, Macrophage inflammatory proteins; RANTES, Regulated upon Activation, Normal T Cell Expressed and Presumably Secreted; TNF, tumor necrosis factor; EPO, Erythropoietin; IFNβ-1, Interferon beta; MDC, Macrophage-Derived Chemokine; TARC, thymus- and activation-regulation chemokine; TIMP-1, Tissue inhibitor of metalloproteinases-1. *C*, Flow cytometric analysis of lung and spleen cells on days 6 and 31 (5 mice per group; 2-way analysis of variance [ANOVA]). *P < .05; **P < .01; ***P < .001 (bars represent mean values). (See gating strategy presented in Supplementary Figure 2 and 3.) Abbreviations: TCM, central memory T cells; TEM, effector memory T cells; T_reg_, regulatory T cells. *D*, Anti-spike immunoglobulin G (IgG) antibody levels present in serum samples collected from noninfected mice (n = 5); infected mice treated with NL-CVX-1 treated on days 10 (n = 4), 21 (n = 4), and 31 (n = 3); and mice infected with 10 PFUs and treated with vehicle (n = 5) (mean with standard deviation; 1-way ANOVA). *P < .05; **P < .01; ***P < .001.

To investigate whether NL-CVX1 would affect immune responses, we evaluated systemic cytokine and antibody responses, as well as immune cell populations in lungs and spleens of mice (Figure 5*A*). Owing to the severity of the infection model used here, for day 31analysis we included a control group in which mice were inoculated with 10 PFUs and administered vehicle.

The cytokine profiles of noninfected mice and NL-CVX1–treated mice were very similar, except for macrophage-derived chemokine (MDC) and monokine induced by interferon (IFN) γ (MIG), interleukin 15, and IFN-β1 (Figure 5*B*), which exhibited increased expression in NL-CVX1–treated mice on day 6. MDC and MIG are cytokines involved in T-cell recruitment: MIG leads the recruitment and activation of proinflammatory T-helper type 1 cells, and MDC recruits anti-inflammatory T-helper type 2. In patients with COVID-19, increased levels of MDC were associated with better prognosis. In contrast, increased MIG levels were associated with increased disease severity and worst prognosis [14, 15], and thus the increase observed in NL-CVX1–treated mice is not fully understood. The inflammatory cytokines interleukin 15 and IFN-β1 have been shown to inhibit viral spread [16, 17] and have been suggested as potential anti- SARS-CoV-2 treatments [18, 19]. Vehicle-treated infected mice displayed increased levels of proinflammatory cytokines, including interleukin 1β, IFN-γ, tumor necrosis factor α, chemokine monocyte chemoattractant protein 1, and granulocyte and granulocyte-macrophage colony-stimulating factor. This expression pattern was absent in NL-CVX1–treated infected mice, suggesting that NL-CVX1 prevented the development of a severe inflammatory response.

Flow cytometric analysis of lung and spleen immune cell populations on day 6 showed increased levels of inflammatory cells, such as neutrophils, activated CD4^+^ (CD69^+^ CD4) and CD8^+^ (CD69^+^ CD8) T cells, and immunosuppressive regulatory T cells, in vehicle-treated but not in NL-CVX1–treated mice (Figure 5*C*). Conversely, reductions in pulmonary B cells, circulating monocytes, and CD4^+^ T cells were observed in vehicle-treated but not in NL-CVX1–treated mice (Figure 5*C*). Because reductions in T and B cells are associated with increased severity of SARS-CoV-2 infection [20, 21], this observation also suggests that NL-CVX1 treatment protects mice from severe disease. Supporting this hypothesis, NL-CVX1–treated mice inoculated with 10^4^ PFUs had fewer naive CD8 T cells in the lungs and naive CD4 T cells in the spleen, and more splenic effector memory CD4 T cells, compared with noninfected mice. In addition, NL-CVX1–treated mice inoculated with 10^4^ PFUs were no different than those inoculated with 10 PFUs, which caused less severe infection (Figure 5*C*).

Regarding antibody response, immunoglobulin G antibodies against spike protein in infected mice treated with NL-CVX1 peaked on day 21, at an average of 3.6-fold above baseline (Figure 5*D*). On day 31, anti-SARS-CoV-2 antibody titers were indistinguishable from those in noninfected mice. On the other hand, mice inoculated with 10 PFUs showed increased levels of anti-SARS-CoV-2 antibodies on day 31, suggesting that NL-CVX1 treatment greatly controls infection and reduces antibody production (Figure 5*D*). We investigated whether antibodies present in infected mice treated with NL-CVX1 could bind to the RBD of ancestral SARS-CoV-2 and the Omicron VOC. By microfluidic diffusional sizing [22, 23] (Supplementary Methods 6), we observed that all animals developed antibodies binding the ancestral SARS-CoV-2 RBD, and 2 of 4 exhibited cross-reactivity to the Omicron RBD (Supplementary Figure 4).

### Protection to Reinfection in Mice Administered with a Single Prophylactic Dose of NL-CVX1

**Figure 6.**
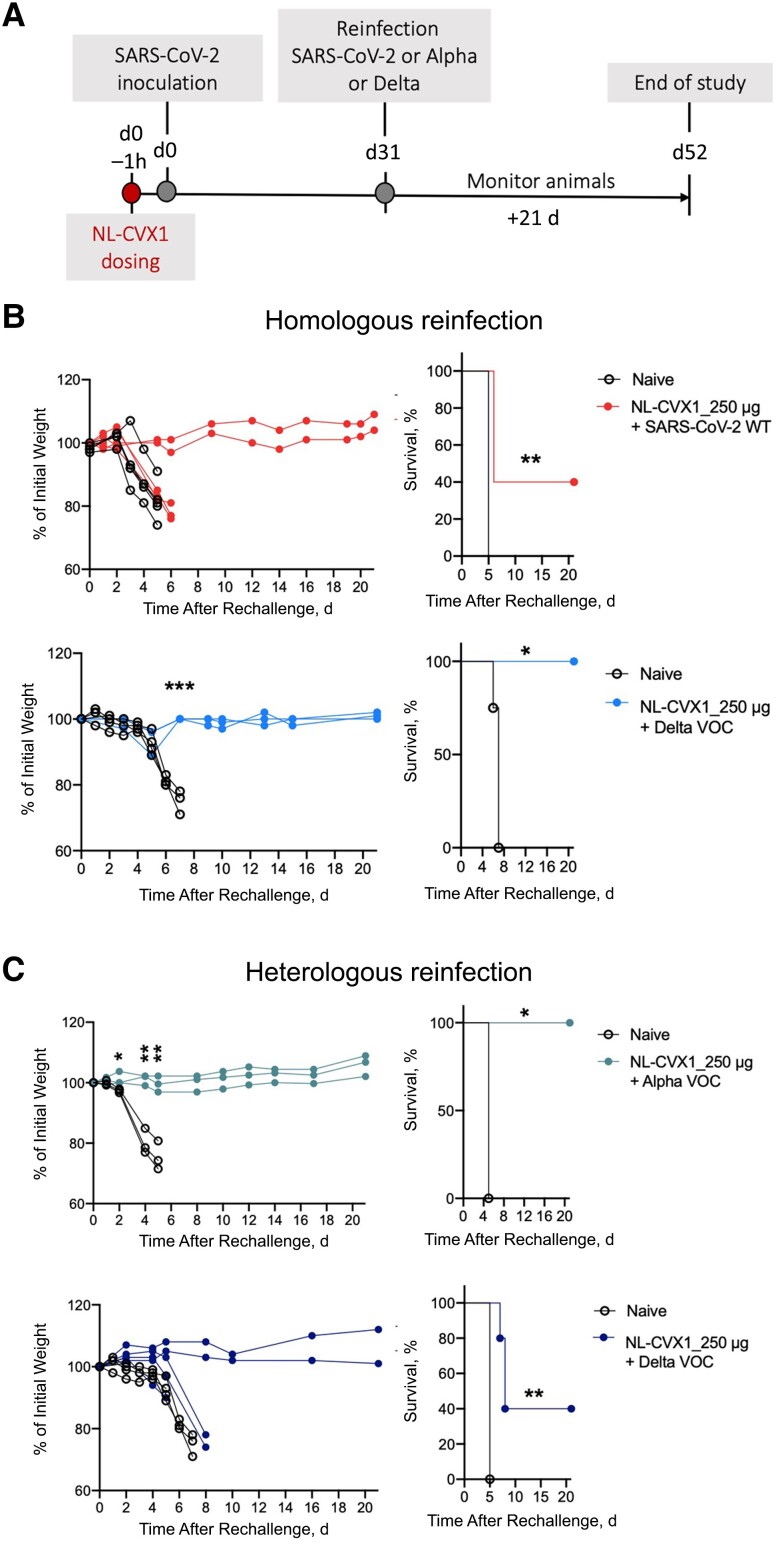
Mice administered with a single prophylactic dose of NL-CVX1 show protection to reinfection. *A*, Female K18– human angiotensin-converting enzyme 2 (hACE2) mice, aged 7–11 weeks, were intranasally administered NL-CVX1 at 250 μg or vehicle, 1 hour (1h) before intranasal inoculation with 10^4^ plaque-forming units (PFUs) of ancestral severe acute respiratory syndrome coronavirus 2 (SARS-CoV-2) or the Delta variant of concern (VOC). On day 31 (d31_ after infection, mice were reinfected with the same virus used for initial infection (homologous reinfection) or reinfected with a different variant (heterologous reinfection). Naive control mice (n = 3; *clear circle*) were never exposed to virus before and were infected for the first time with the same virus variant as the one used for reinfection. Mice were monitored for body weight loss, disease, and death for 21 days. *B*, Body weight change and percentage of survival in mice administered NL-CVX1 after homologous reinfection with ancestral SARS-CoV-2 virus (n = 5) or the Delta VOC (n = 5). *C*, Body weight change and percentage of survival of mice, initially infected with ancestral SARS-CoV-2 (wild type [WT]) and administered NL-CVX1, after heterologous reinfection with the Alpha (n = 3) or the Delta (n = 5) VOC. Body weight changes in experimental groups were statistically compared using multiple *t* tests, and survival rates were compared using Mantel-Cox tests. *P < .05; **P < .01; ***P < .001.

We then investigated whether NL-CVX1–treated mice would be protected from a second infection. Mice treated with 250 μg of NL-CVX1 were inoculated with ancestral SARS-CoV-2 or the Delta VOC and reinfected 31 days later with the same virus as the first inoculation (homologous reinfection) or a different variant, such as the Alpha or Delta VOCs (heterologous reinfections) (Figure 6*A*). Naive mice, which were never exposed to the virus or to NL-CVX1, were administered vehicle and infected with the same variant used for reinfections (Figure 6*B* and 6*C*). As expected, naive mice reached humane end points on day 5 (Figure 5*B* and 5*C*). In homologous reinfection with ancestral SARS-CoV-2, 3 of 5 mice showed body weight loss and reached humane end points on day 6 after reinfection (40% survival rate) (Figure 6*B*). However, in homologous reinfection with the Delta VOC, mice were fully protected from reinfection (100% survival rate). Interestingly, in heterologous reinfections with the Alpha VOC, in which mice were initially infected with ancestral SARS-CoV-2, no body weight loss nor signs of disease were observed for 21 days after reinfection (100% survival rate) (Figure 6*C*), while 3 of 5 mice initially infected with ancestral SARS-CoV-2 and reinfected with the Delta VOC showed body weight loss and succumbed to infection by day 8 after reinfection (40% survival rate) (Figure 6*C*).

### Postexposure Therapeutic Activity of NL-CVX1 in Mice Infected with SARS-CoV-2

**Figure 7.**
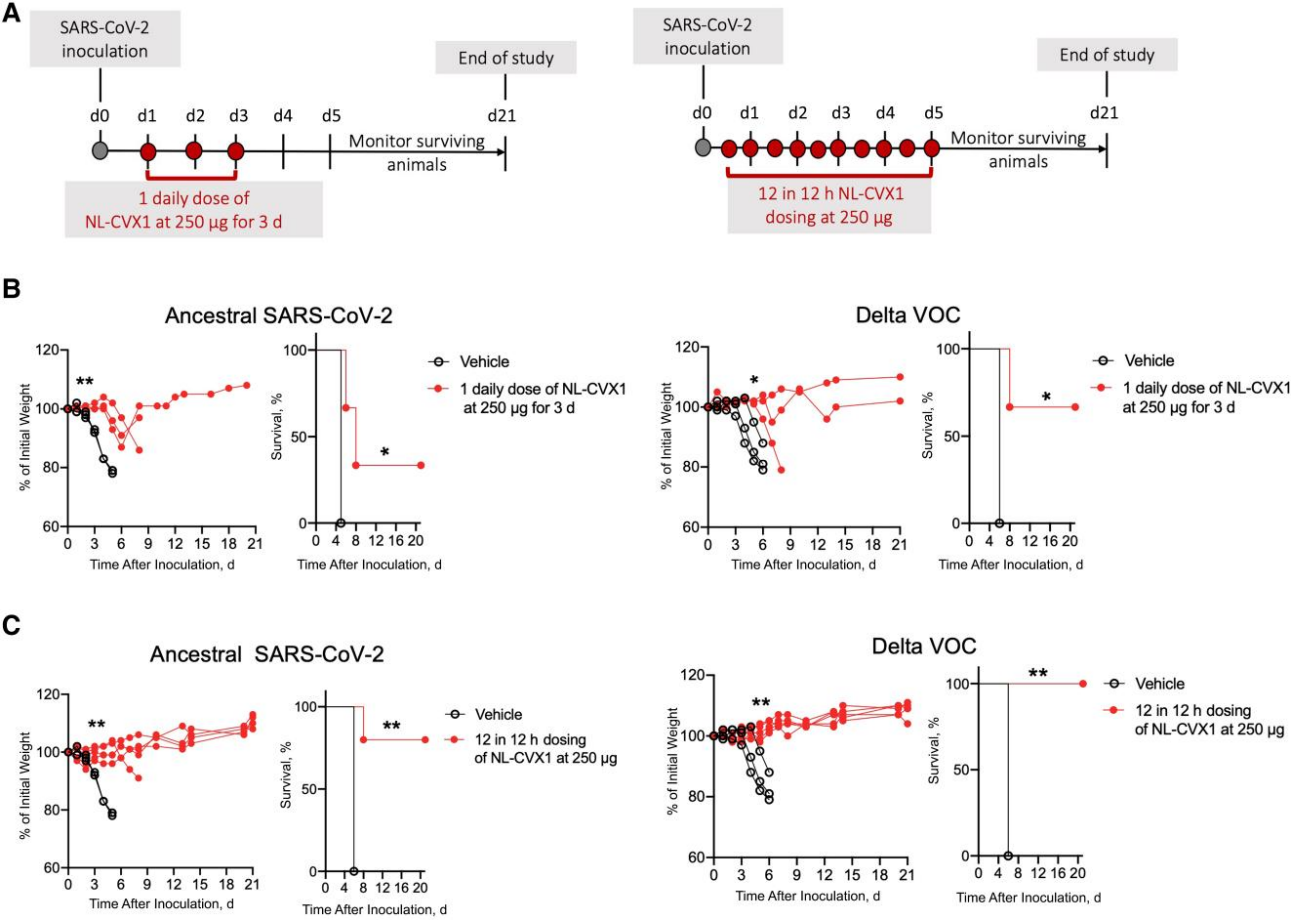
Postexposure therapy with NL-CVX1 reduces severe disease in mice infected with ancestral severe acute respiratory syndrome coronavirus 2 (SARS-CoV-2) virus and the Delta variant of concern (VOC). *A*, Postexposure therapy study design. Female K18– human angiotensin-converting enzyme 2 mice, aged 7–11 weeks, were intranasally administered NL-CVX1 at 250 μg or vehicle on days 1, 2, and 3 after infection or every 12 hours for 5 days. Mice were monitored for weight loss, disease, and death for 21 days. *B*, Body weight change and percentage of survival in mice infected with ancestral SARS-CoV-2 or the Delta VOC after administration of vehicle (*clear circle*; n = 3) or 3 doses of NL-CVX1 250 μg for 3 days (*filled circle*; n = 3). *C*, Body weight change and percentage of survival in mice infected with ancestral SARS-CoV-2 or the Delta VOC after administration of vehicle (*clear circle*; n = 3) or NL-CVX1 250 μg (*filled circle*; n = 5) every 12 hours after infection, for 5 days. Body weight changes were statistically compared using multiple *t* tests, and survival rates were compared using Mantel-Cox tests. *P < .05; **P < .01.

To investigate the therapeutic potential of NL-CVX1, we tested 2 dosing schemes (Figure 7*A*). In mice infected with ancestral SARS-CoV-2 and administered 1 daily dose of NL-CVX1 for 3 days, a 2-day delay in body weight loss was observed. On day 5, vehicle-treated mice had an average weight loss of 21% versus 6% in NL-CVX1–treated mice (Figure 7*B*, *left panel*). In mice infected with Delta VOC and administered 1 daily dose of NL-CVX1 for 3 days, significant weight loss was observed in 1 of 3 mice starting on day 6 (Figure 7*B*, *right panel*). Survival rates of 33% and 67% were observed for mice infected with ancestral SARS-CoV-2 or with the Delta VOC, respectively (Figure 7*B*).

NL-CVX1 administration every 12 hours after infection for 5 days increased protection levels against severe disease (Figure 7*C*), with significant body weight loss observed in only 1 mouse infected with ancestral SARS-CoV-2 (Figure 7*C*, *left panel*). Survival rates of 80% and 100% were observed for mice infected with ancestral SARS-CoV-2 or with the Delta VOC, respectively (Figure 7*C*).

## DISCUSSION

Since the emergence of SARS-CoV-2, means to prevent virus entry by interfering with the binding to the host cell receptor have been investigated. Monoclonal antibodies are the most common approach [24]. However, their use at early stages of disease in nonhospitalized settings is limited by their administration route (intravenous infusion), and their large size, which hampers tissue penetration. Novel approaches based on computational designed proteins may overcome these limitations [8, 25–27]. Examples of these proteins are NL-CVX1 and LBC1, which prevent SARS-CoV-2 host cell invasion by binding to the RBD of spike protein. LCB1 is a 56–amino acid miniprotein that showed promising results in preclinical testing [25, 26], but the Omicron VOC was reported to be resistant to LCB1 [28].

Here, we established the preclinical efficacy of NL-CVX1 against SARS-CoV-2 in K18-hACE2 mice. We demonstrated that NL-CVX1 prevented mice from developing severe disease, when administered prophylactically or therapeutically. While prophylactic administration conferred full protection (100% survival), postexposure therapy with a daily dose of NL-CVX1 for 3 days decreased survival to 33% for ancestral SARS-CoV-2 and 67% for the Delta VOC. More frequent dosing of NL-CVX1 every 12 hours for 5 days increased survival to 80% for ancestral SARS-CoV-2 and 100% for the Delta VOC. Fine tuning of NL-CVX1 dosing allows protection from severe disease, yet further pharmacokinetics studies are needed to determine the optimal therapeutic dosing scheme. Because the K18-hACE2 model used in this study is highly susceptible to SARS-CoV-2 and vehicle-treated mice develop severe disease and reach humane end points within 5 to 7 days after inoculation [9], the treatment window in this model is very reduced. Inoculation with lower viral titers could be considered, but the only inoculation dose that did not cause severe disease was of 10 PFUs. However, it led to such mild infection that is virtually indistinguishable from noninfected mice (Supplementary Methods 8 and Supplementary Figure 1).

Interestingly, we observed that infected mice administered with NL-CVX1 were protected from reinfection a month after administration. In humans, it was reported that infection with any SARS-CoV-2 variant is highly effective at preventing severe and fatal COVID-19 after reinfection [29, 30]. Protection against reinfection is achieved by the development of cellular and humoral memory. In the current study, we observed that mice treated with NL-CVX1 were able to develop immunological memory, through the differentiation of effector memory CD4 T cells and production of anti-SARS-CoV-2 antibodies. However, on day 31—when mice are reinfected—NL-CVX1–treated mice showed a decrease in antibodies levels (comparable to noninfected mice), increased levels of memory T cells were observed, possibly contributing to the protection against reinfection.

NL-CVX1 activity against Delta and Omicron infection was also investigated. Higher levels of viral RNA were detected in the lungs of mice infected with the Omicron VOC and treated with NL-CVX1, compared with mice infected with ancestral SARS-CoV-2 or the Delta VOC. We believe that the extensive spike protein mutations present in the Omicron VOC, which are known to impact transmissibility and immune evasion [31], may increase the affinity of the Omicron VOC toward hACE2, therefore attenuating NL-CVX1 blocking capacity. Further investigation of higher doses or multidosing schemes is required to ultimately determine the efficacy of NL-CVX1 against the Omicron VOC.

In conclusion, we show the role of NL-CVX1 as a potent anti-SARS-CoV-2 treatment approach. NL-CVX1 has several advantages over antibody-based therapeutics, such as (1) intranasal administration, which facilitates self-administration and increases patient compliance; (2) high and specific binding affinity that translates into effective neutralization of SARS-CoV-2; (3) cost-effective manufacturing; and (4) high stability, thereby enabling simplified transport and storage. Further testing of different routes of administration, such as nebulization, and evaluation of immunogenicity, pharmacokinetics, and pharmacodynamics in larger mammals is warranted to guide robust clinical evaluation in future phase I clinical trials.

## Supplementary Data

Supplementary materials are available at *The Journal of Infectious Diseases* online. Consisting of data provided by the authors to benefit the reader, the posted materials are not copyedited and are the sole responsibility of the authors, so questions or comments should be addressed to the corresponding author.

## Supplementary Material

jiad135_Supplementary_Data

## Notes

***Acknowledgments*.** We acknowledge BEI Resources, National Institute of Allergy and Infectious Diseases, National Institutes of Health, for providing the viral variants, and South Africa's National Institute for Communicable Diseases of the National Health Laboratory Service for provided the biological material to the World Health Organization BioHub System. We thank Andreia F. Mosca and Claudia Fonseca for virology expertise, and all staff from iMM's Flow Cytometry Unit, Comparative Histopathology Unit, and rodent and biosafety level 3 laboratory facilities. This project has received funding from the European Union's Horizon 2020 research and innovation programme under grant agreements number 852985.

***Author contributions*.** M. R., L. T., D. A. S., D. S., and G. J. L. B. designed the experiments. M. R, C. T., A. R. C., and M. P. d. M. conducted the mouse studies. M. R, C. T., A. R. C. and M. P. d. M. performed viral load quantification by plaque assays. C. T. and A. R. C performed polymerase chain reaction analysis. M. R. and C. L. A. conducted flow cytometry measurements and immune cell characterization data analysis. A. G and M. V. developed and conducted enzyme-linked immunosorbent assays and analysis. M. M. S., H. A., and T. P. J. K. performed microfluidic antibody affinity profiling. P. R. and T. C. performed histopathological analysis. M. R. and G. J. L. B wrote the manuscript, with input from all other authors.

***Financial support.*** This work was supported by Neoleukin Therapeutics.
