# Supplementary material for: De Novo Human Angiotensin-Converting Enzyme 2 Decoy NL-CVX1 Protects Mice From Severe Disease After Severe Acute Respiratory Syndrome Coronavirus 2 Infection
Source: J Infect Dis. 2023 Jun 5;228(6):723–33. doi: 10.1093/infdis/jiad135 (PMC10503951; doi:10.1093/infdis/jiad135)
Supplement: jiad135_Supplementary_Data [file jiad135_supplementary_data.zip › SI_revised.pdf]

Supplementary Material

Supplementary Figures

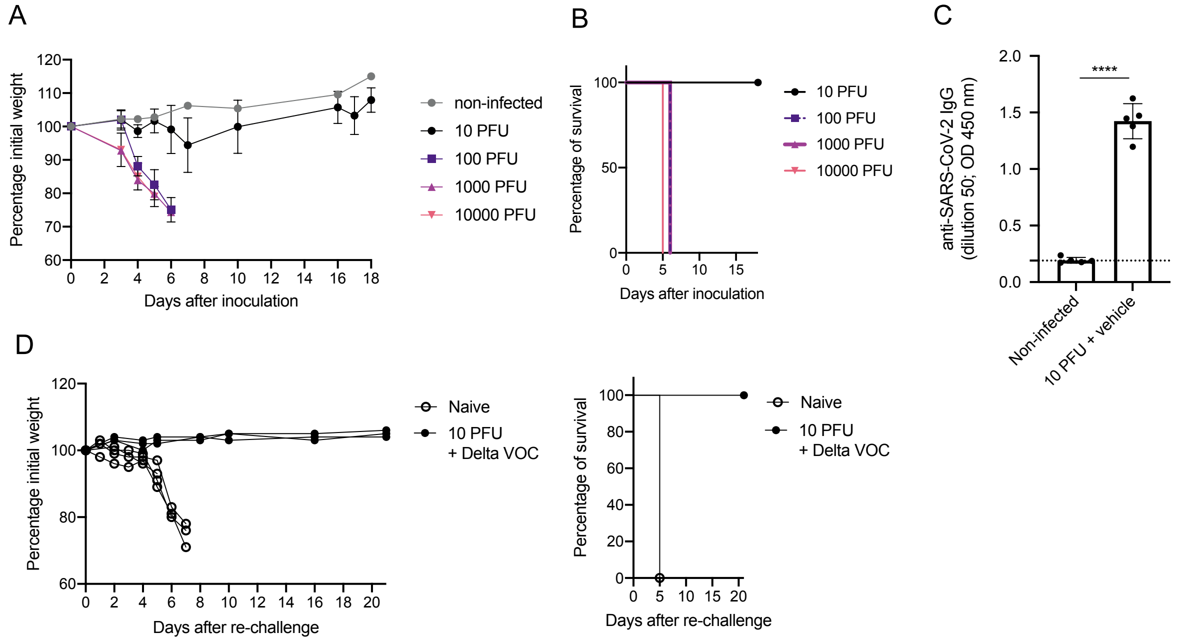

Supplementary Figure 1. Inoculation of K18hACE mice with different viral loads of ancestral SARS-CoV-2 and re-infection of 10PFU inoculated mice with delta VOC. Body weight monitoring **(A)** and percentage of survival **(B)** of seven- to eleven-week-old K18hACE2 non-infected mice (n = 5) and of mice that were intranasally inoculated with 10 (n = 2), 100 (n = 2), 1000 (n = 2) and 10000 (n = 2) PFU. Mice inoculated with 100, 1000 and 10000 PFU reach humane endpoints between Day 5 and 6. Mice inoculated with 10 PFU did not show body weight loss nor disease signs and have 100% survival. **(C)** Anti-spike IgG antibody levels present in serum samples collected from non-infected (n = 5) and from mice inoculated with 10 PFU and administered with vehicle (n = 5) on Day 31 post-infection (mean ± SD; unpaired t-test, ****P value < 0.0001). **(D)** Body weight monitoring and percentage of survival K18hACE2 mice (n = 6) initially inoculated with 10 PFU of ancestral SARS-CoV-2 and re-infected with 0.5 x 10^4 PFU of delta VOC (n = 3, filled circles) on Day 31 post-infection. Naive control mice (n = 3, empty circles) correspond to animals that were never exposed to virus before and were infected for the first time with the same virus variant as the one used for re-infection. During 21 days of monitoring mice initially inoculated with 10 PFU (filled circles) did not show body weight loss nor disease signs and had 100% survival rate after re-infection.

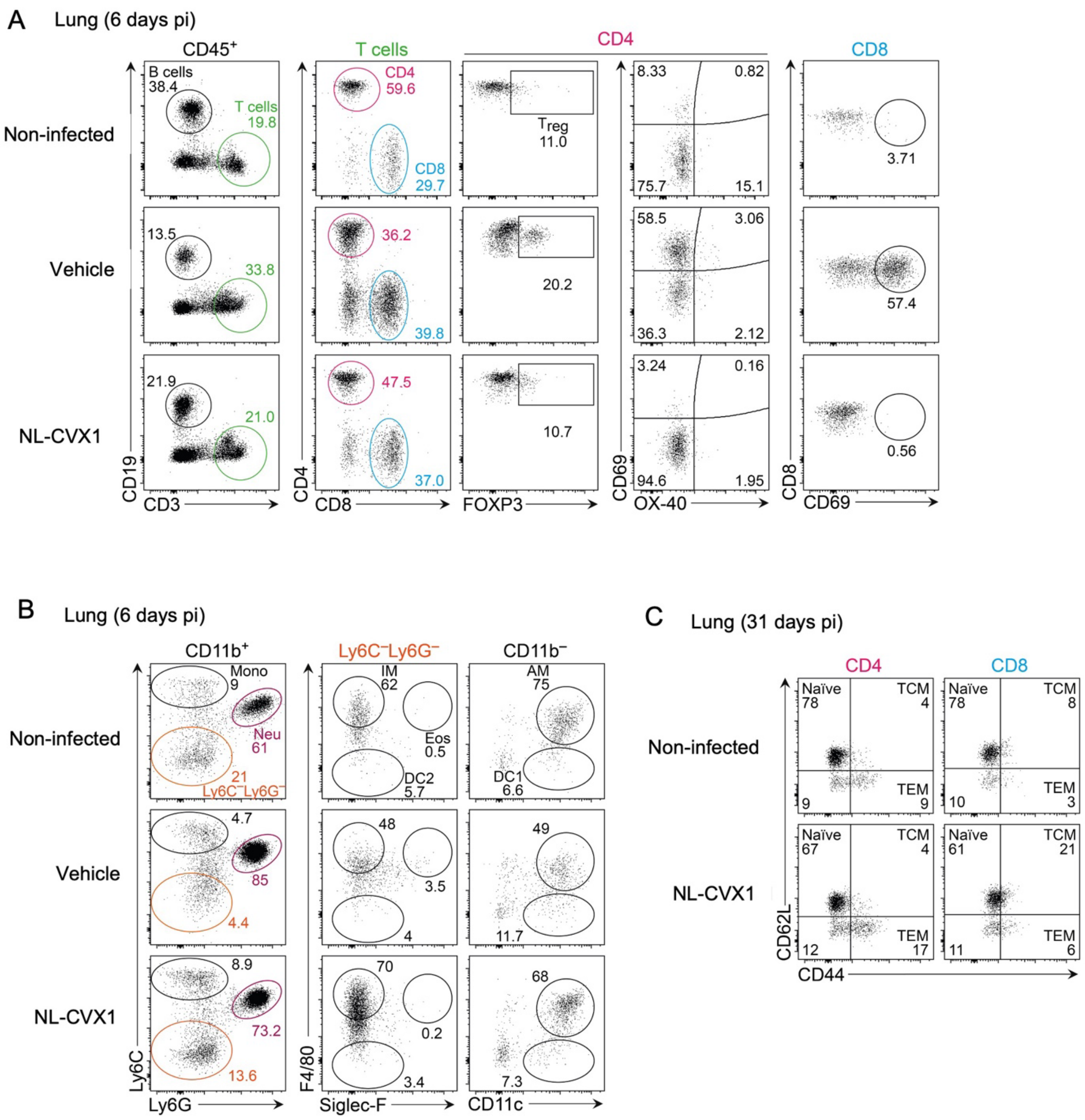

Supplementary Figure 2. Gating strategy used in the flow cytometric analysis of lung inflammatory cells. **(A)** Representative FACS plots and corresponding gating strategy used for lymphoid cells obtained from lung homogenates on Day 6. **(B)** Gating strategy for lung myeloid cells on Day 6. **(C)** Evaluation of memory T cells in the Lungs on Day 31. Treg: Regulatory T cells; Mono: Monocytes; Neu: Neutrophils; IM: Interstitial Macrophages; Eos: Eosinophils; DC2: Type 2 Dendritic Cells; AM: Alveolar Macrophages; DC1: Type 1 Dendritic Cells; TCM: Central Memory T cells; TEM: Effector Memory T cells.

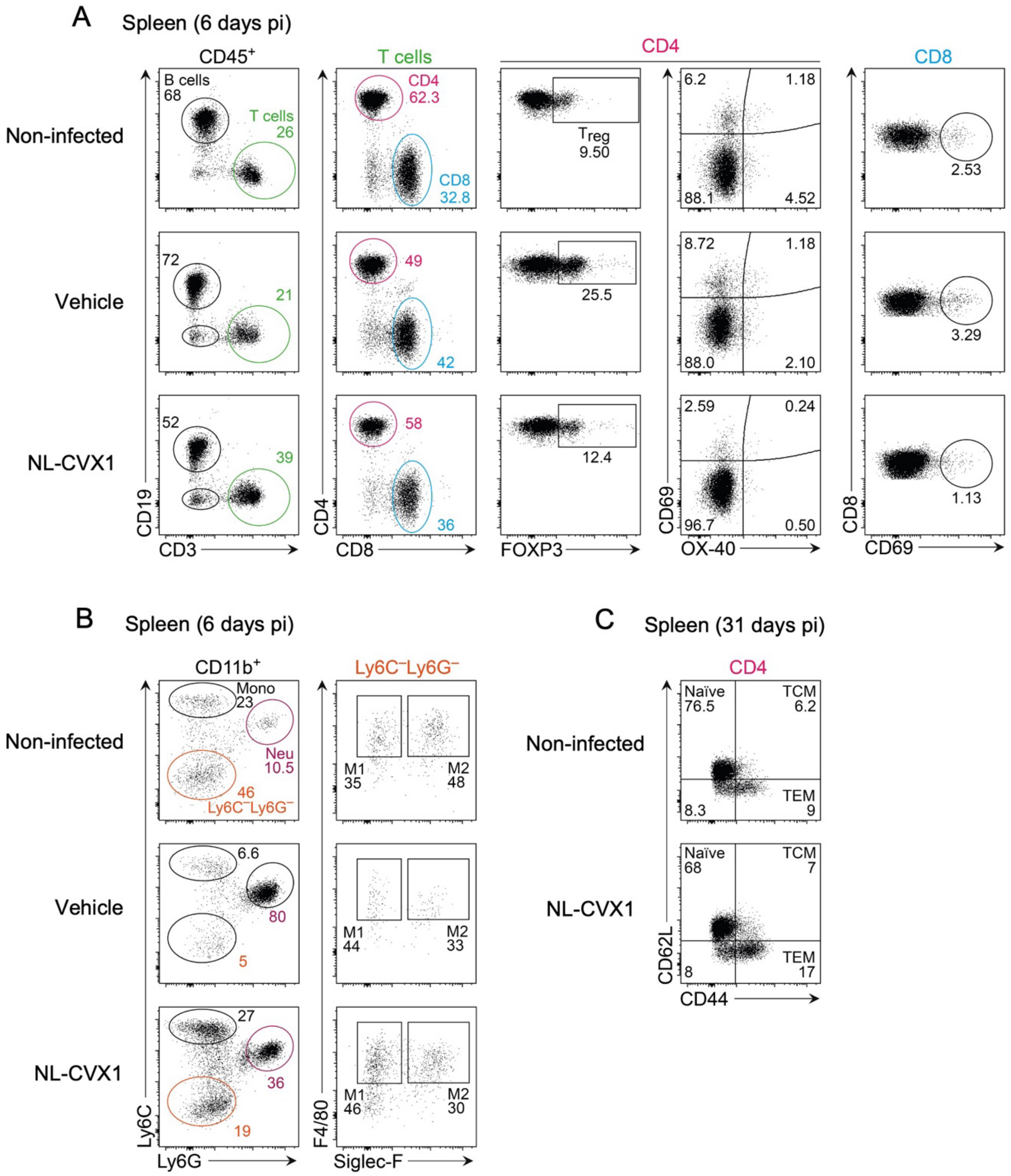

Supplementary Figure 3. Gating strategy used in the flow cytometric analysis of spleen inflammatory cells. **(A)** Representative FACS plots and corresponding gating strategy used for lymphoid cells isolated from the spleen on Day 6 pi. **(B)** Gating strategy for splenic myeloid cells on Day 6 pi. **(C)** Evaluation of memory T cells in the spleen on Day 31 pi. Treg: Regulatory T cells; Mono: Monocytes; Neu: Neutrophils; M1: Type 1 Macrophages; M2: Type 2 Macrophages; TCM: Central Memory T cells; TEM: Effector Memory T cells.

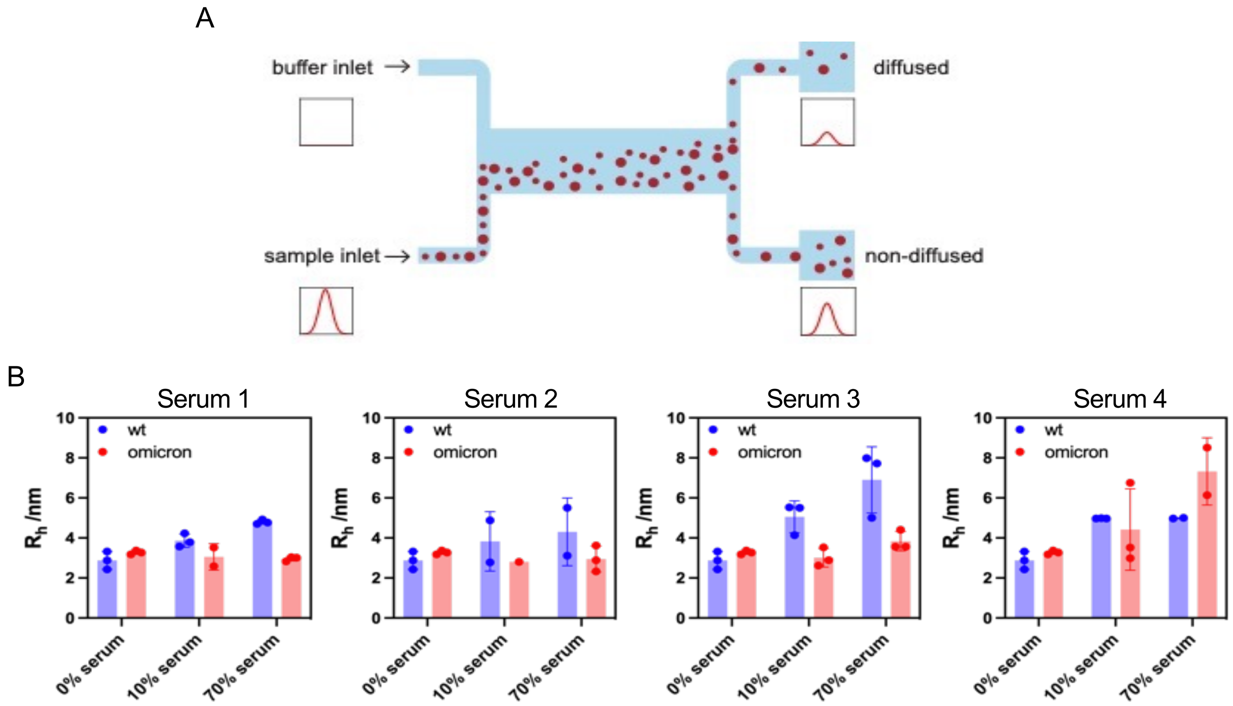

Supplementary Figure 4. Antibody affinity against the receptor binding domain of wild-type SARS-CoV-2 and omicron variant. **(A)** A stream of sample and buffer co-flows in a microfluidic chip, so that mixing occurs by diffusion only. The extent of diffusion from the sample stream to the buffer stream is detected by measuring the intensity of both halves of the chamber at the end of the channel. **(B)** Hydrodynamic radius of two variants of labelled SARS-CoV-2 RBD (wild-type in blue and omicron in red) in presence of different serum concentrations. An increase in radius at increased serum concentration shows binding. Serum 1 and serum 2 shows significant binding against wild-type but not omicron. Serum 3 has significant binding against wild-type and a light cross-reactivity against omicron. Serum 4 shows a high binding response against both wild-type and omicron.

Supplementary Methods

Supplementary methods 1 - Lung homogenization

The left lung was homogenized using ULTRA-TURRAX® Tube Drive control. After homogenization (4 minutes), lung homogenates were centrifuged at 1000 rpm (203x g) for 2 minutes. Supernatant was obtained for plaque assay and added to RLT lysis buffer (Qiagen) for PCR analysis.

Supplementary methods 2 - Primer sequences for viral load quantification by PCR

Primer for envelope (E) gene, nucleocapsid (N) gene, and 18S (NZYTech, Lisbon, Portugal) was used to amplify target genes. The sequences of the primers are as follow: E gene (F: 5'-ACAGGTACGTTAATAGTTAATAGCGT-3'; R: 5'-ATATTGCAGCAGTACGCACACA-3'); N gene (F: 5'-GACCCCAAAATCAGCGAAAT-3'; R: 5'-TCTGGTTACTGCCAGTTGAATCTG-3'); 18S (F: 5'-GTAACCCGTTGAACCCCATT-3'; R: 5'-CCATCCAATCGGTAGTAGCG-3').

Supplementary methods 3 - Histopathology evaluation criteria

Qualitative and quantitative evaluation of infected lung tissues followed previously published guidelines (Meyerholz DK, Beck AP. Histopathologic Evaluation and Scoring of Viral Lung Infection. *Methods Mol Biol*. 2020;2099:205-220. doi:10.1007/978-1-0716-0211-9_16; Zheng J, Wong LR, Li K, et al. COVID-19 treatments and pathogenesis including anosmia in K18-hACE2 mice. *Nature*. 2021;589(7843):603-607. doi:10.1038/s41586-020-2943-z). The following criteria was used for scoring edema, hyaline membrane formation and necrotic cellular debris in the lung: 0- none; 1- uncommon detection in <5% lung fields (200x); 2- detectable in up to 33% of lung fields; 3- detectable in up to 33-66% of lung fields; 4- detectable in >66% of lung fields. For scoring neutrophil infiltration: 0- within normal limits; 1-scattered polymorphonuclear neutrophils (PMNs) sequestered in septa; 2- #1 plus solitary PMNs extravasated in airspaces; 3-#2 plus small aggregates in vessel and airspaces. For scoring mononuclear infiltrates, thrombosis, and hemorrhage: 0-none; 1- uncommon detection in <5% lung fields (200x); 2- detectable in up to 33% of lung fields; 3- detectable in up to 33-66% of lung fields; 4- detectable in >66% of lung fields. Results, for individual animals, are given as sum of all scores.

Supplementary methods 4 – Lung and spleen sell isolation for inflammatory cell staining

Right lung was digested in 0.5 μg/mL of Collagenase D and 10 μg/mL of DNAse (Roche) and incubated for 30 minutes at 37 ºC. Lung and spleen were macerated and cells were washed in complete media. Cell pellet was incubated with RBC Lysis Buffer (BioLegend) at 1X and centrifuged at 500x g, for 5 minutes at room temperature (RT). $2x10^6$ cells were incubated with Fc Block (1:100) in PBS, for 20 minutes at 4ºC, washed in FACS buffer by centrifuging at 620x g, for 2 minutes, at RT and then resuspended in FACS buffer until flow cytometry analysis was performed.

Supplementary methods 5 - Extracellular antibody mix for staining of inflammatory cells

All antibodies were purchased from BioLegend unless stated otherwise. The following antibody mix was used to stain lung and spleen inflammatory cell populations:

CD45 (clone 30-F11), F4/80 (BM8), CD11c (N418), Ly6C (HK1.4), Ly6G (1A8-Ly6G), CD4 (GK1.5), CD3 (17A2), CD8a (53-6.7), CD11b (M1/70), MHC II (M5/114.15.2), TER119, CD19 (1D3/CD19), CD69 (H1.2F3), OX-40 (OX-86), Siglec-F (S17007L) and CD62L (MEL-14). CD49b (DX5), Foxp3 (FJK-16s) and eBioscience Fixable Viability Dye eFluor 78 were obtained from Ebiosciences and CD44 (IM7) from Merck.

Table S1: Details of antibodies used for staining of inflammatory cells.

**Antibody**	**Fluorophores**	**Concentration or Dilution**	**Brand**
CD45 (clone 30-F11)	BV510	1:100	BioLegend
F4/80 (BM8)	PE-Cy7	1:100	BioLegend
CD11c (N418)	BV711	1:200	BioLegend
Ly6C (HK1.4)	FITC	1:100	BioLegend
Ly6G (1A8-Ly6G)	BV605	1:200	BioLegend
CD4 (GK1.5)	PE-Cy7	1:500	BioLegend
CD4 (GK1.5)	BV605	1:200	BioLegend
CD3 (17A2)	BV711	1:100	BioLegend
CD8a (53-6.7)	PerCP	1:400	BioLegend
CD8a (53-6.7)	BV605	1:500	BioLegend
CD11b (M1/70)	BV421	1:400	BioLegend
MHC II (M5/114.15.2)	PerCP	1:200	BioLegend
TER119	APC	1:100	BioLegend
CD19 (1D3/CD19)	APC	1:200	BioLegend
CD69 (H1.2F3)	PE-Cy7	1:200	BioLegend
OX-40 (OX-86)	BV421	1:100	BioLegend
Siglec-F (S17007L)	PE	1:100	BioLegend
CD62L (MEL-14)	FITC	1:100	Ebiosciences
CD49b (DX5)	FITC	1:100	Ebiosciences
Foxp3 (FJK-16s)	PE	1:50	Ebiosciences
Fixable Viability Dye eFluor 78	eFluor 780	1:1000	Ebiosciences
CD44 (IM7)	Pacific Blue	1:500	Merck

Supplementary methods 6 - Cytokine and chemokine assay biomarkers

Heat inactivated sera was shipped to Eve Technologies (Canada) and the Mouse Cytokine/Chemokine 31-Plex Discovery Assay® (Luminex immunoassay) was performed for the following biomarkers: Eotaxin, Erythropoietin, 6Ckine, Fractalkine, G-CSF, GM-CSF, IFNB1, IFNγ, IL-1α, IL-1β, IL-2, IL-3, IL-4, IL-5, IL-6, IL-7, IL-9, IL-10, IL-11, IL-12 (p40), IL-12 (p70), IL-13, IL-15, IL-16, IL-17, IL-20, IP-10, KC, LIF, LIX, MCP-1, MCP-5, M-CSF, MDC, MIG, MIP-1α, MIP-1β, MIP-2, MIP-3α, MIP-3B, RANTES, TARC, TIMP-1, TNFα, and VEGF.

Supplementary methods 7 - ELISA for quantification of IgG antibodies

Serum samples were analysed for antibodies using SARS-CoV-2 Spike protein or NL-CVX1 decoy, followed by titer determination. To this end we used our in-house developed protocol. Briefly, flat-bottom 96-well plates (Microlon plates medium binding; Greiner) were coated with recombinant protein Spike (2 µg/mL) or decoy (2 and 5 µg/mL) prepared in PBS (50 µL/well). Plates were blocked with 200 µL/well of 3% nonfat milk powder in PBS, 0.1% Tween-80 for 1 hour at room temperature and then washed with PBS, 0.1% Tween-80 3x as described previously.20 Serum samples were diluted 1:50 in PBS, 0.1% Tween-80 + 1% non-fat milk powder, added to plates (100 µL/well) and incubated for 1 hour at room temperature and washed with PBS, 0.1% Tween-80 3 times. Hereafter horseradish peroxidase (HRP)-labelled anti-mouse IgG (Thermo Fisher) was diluted in PBS, 0.1% Tween-80 and 1% non-fat milk powder (50 µL/well) for 1h at room temperature, washed with PBS, 0.1% Tween-80 3 times, and developed with TMB substrate solution (TMB Substrate Reagent Set, BD OptEIA™, 555214), 100 µL/well for 10 minutes. The reaction was stopped with 2M sulfuric acid (50 µL/well) and optical density at 450 nm was measured via SPARK (TECAN) plate reader. Levels of anti-spike IgG and anti-decoy IgG were quantified by two-fold serial dilutions. Each plate contained positive and negative quality control (QC) samples, composed of a pool of positive and negative samples, respectively.

Supplementary methods 8 - Inoculation of K18-hACE2 mice with different viral loads

K18-hACE2 mice were intranasally inoculated with 50 µL of 10, 100, 1000 and 10000 PFU of ancestral SARS-CoV-2 and monitored at least twice a week for body weight loss, morbidity and mortality for 18 days.

Supplementary methods 9 - Microfluidic Antibody Affinity Profilling (MAAP)

Microfluidic antibody affinity profiling (MAAP) was used to assess antibody concentration and affinity to the receptor binding domain (RBD) of SARS-CoV-2 wild-type virus and omicron variant of concern. Serum samples were collected from four mice infected with SARS-CoV-2 ancestral virus and prophylactically treated with a single dose of NL-CVX1 at 250 µg on Day 21 post infection. For labelling, receptor binding domain of SARS-CoV-2 wild-type and omicron variants were buffer exchanged to 0.1 M $NaHCO_3$ (pH 8.2; Sigma Aldrich, Gillingham, UK) on an Amicon Filter (3 kDa cut-off, Sigma Aldrich, Gillingham, UK). The protein was then incubated with a 4-times molar excess of Alexa Fluor 647 *N*-hydroxysuccinimidyl ester (ThermoFisher Scientific Ltd, Waltham MA, US) for 16 hours at 4°C. Excess dye was removed by size exclusion chromatography on a Superdex 200 10/300 gl increase column (Cytiva, Marlborough, UK) connected to an AKTA pure instrument (Cytiva, Marlborough, UK), with PBS as elution buffer and a flow rate of 0.5 mL/minute. The labelled RBD was stored at -80 °C until further use.

For affinity measurements, pristine serum was incubated with RBD and buffer to yield different serum dilutions. The samples were incubated for 1 hour at room temperature. Then, the hydrodynamic radius was determined on the One M instrument (Fluidic Analytics Ltd, Cambridge, UK). The dissociation constant and antibody concentrations were determined using the relation

$$K_d = \frac{[Ab][RBD]}{[AbRBD]}$$

where $[Ab]$ is the unbound antibody binding site concentration, $[RBD]$ the concentration of the unbound RBD, and $[AbRBD]$ the concentration of the antibody-binding site-RBD-complex, whereby

$$[AbRBD] = \frac{(\alpha[Ab]_{tot} + R_0 + K_d) \pm \sqrt{(\alpha[Ab]_{tot} + R_0 + K_d)^2 - 4\alpha[Ab]_{tot}[RBD]}}{2}$$

using the Cloud service of Fluidic Analytics Ltd (Cambridge, UK).
