# Supplementary figures and images for: De Novo Human Angiotensin-Converting Enzyme 2 Decoy NL-CVX1 Protects Mice From Severe Disease After Severe Acute Respiratory Syndrome Coronavirus 2 Infection
Source: J Infect Dis. 2023 Jun 5;228(6):723–33. doi: 10.1093/infdis/jiad135 (PMC10503951; doi:10.1093/infdis/jiad135)

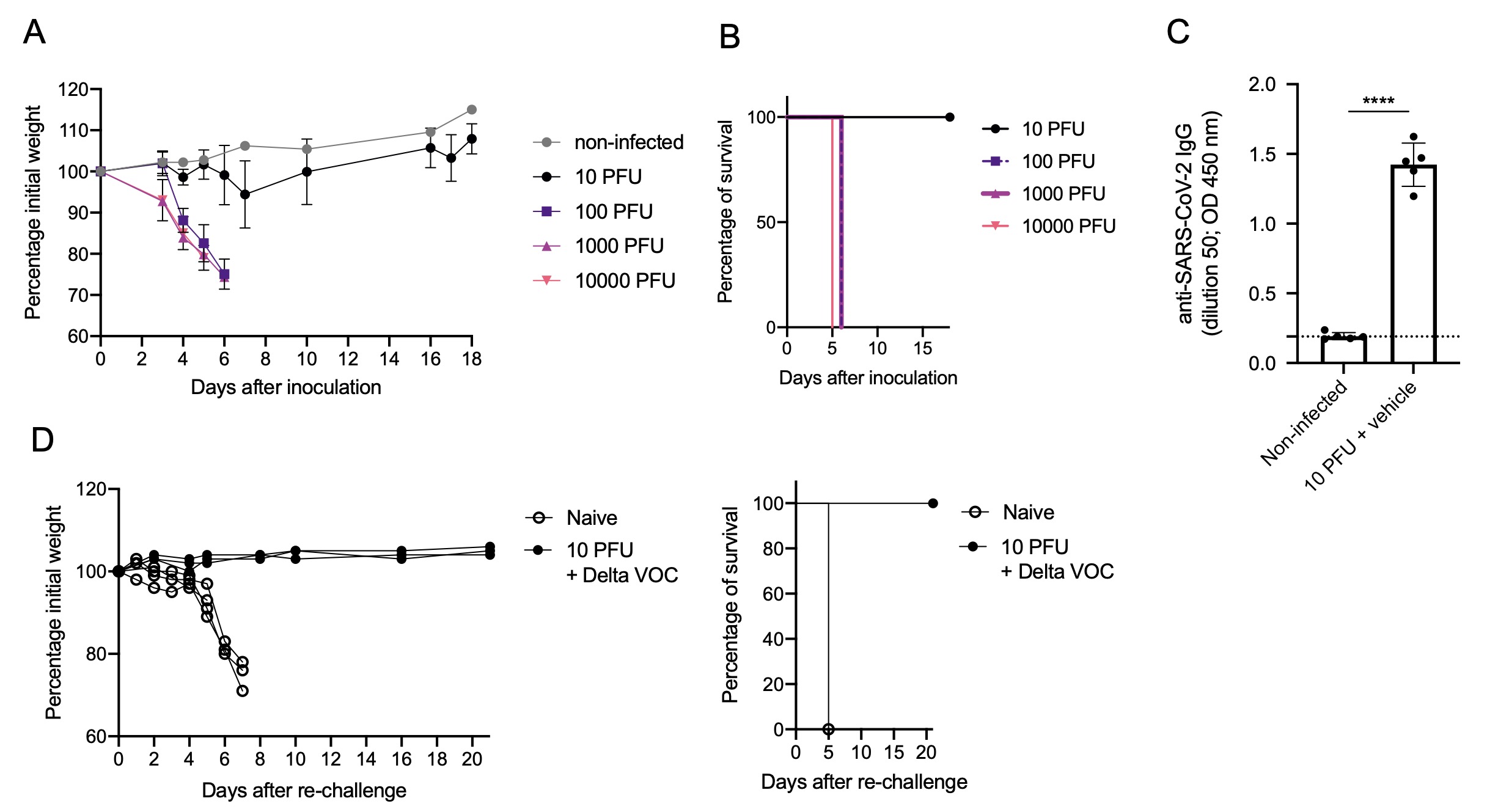

Supplement: jiad135_Supplementary_Data [file jiad135_supplementary_data.zip › FigureS1.jpg]

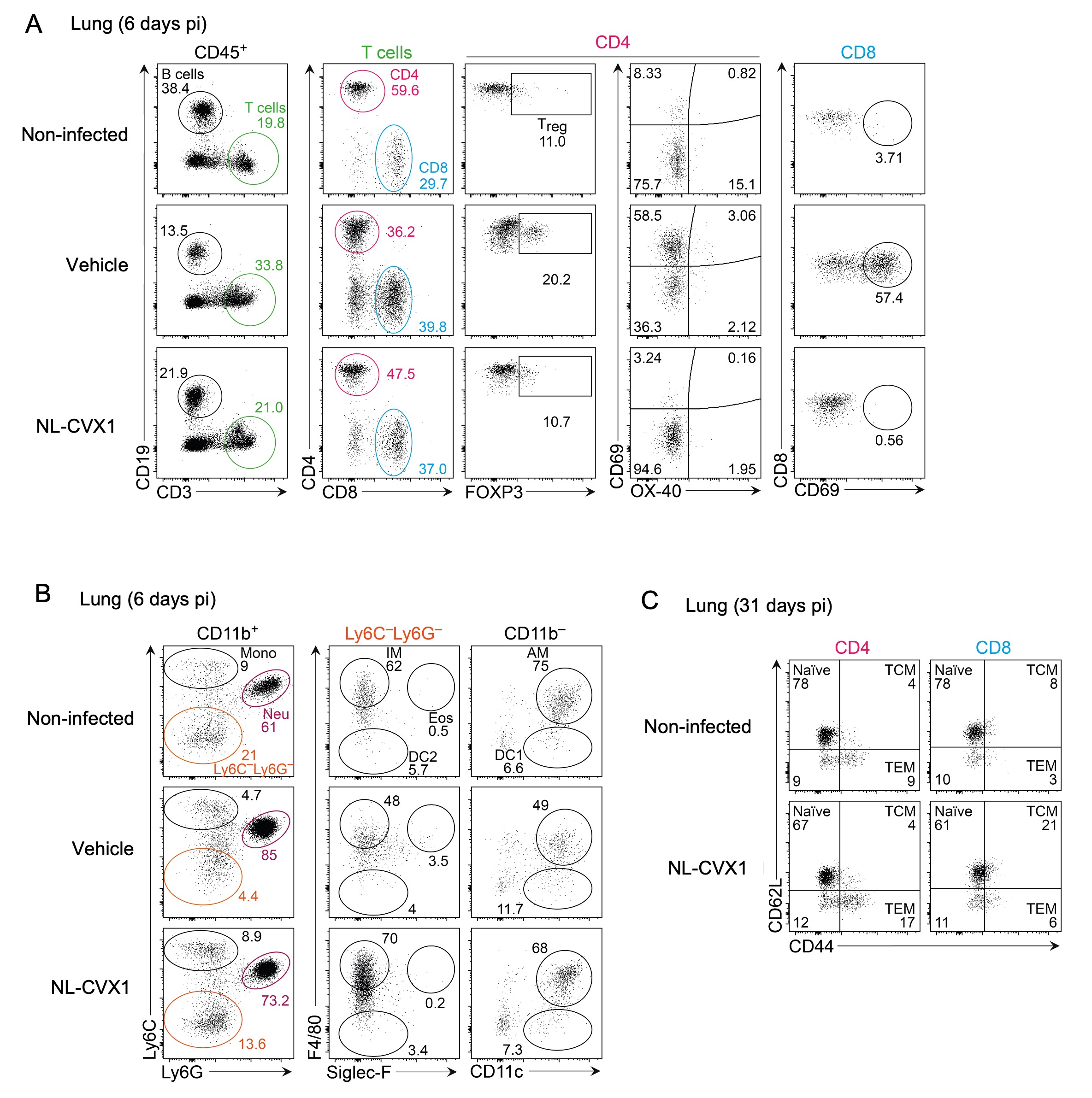

Supplement: jiad135_Supplementary_Data [file jiad135_supplementary_data.zip › FigureS2.jpg]

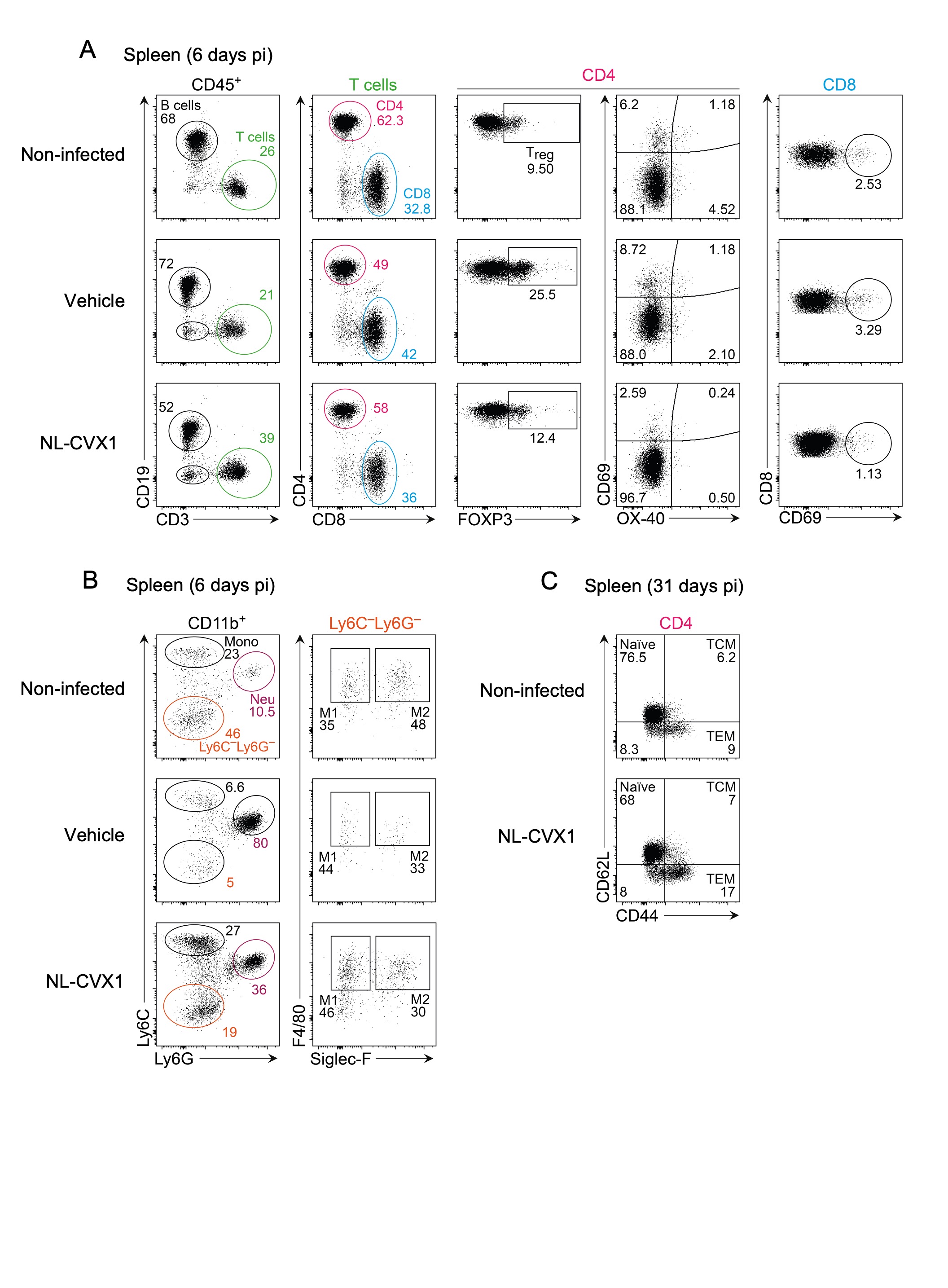

Supplement: jiad135_Supplementary_Data [file jiad135_supplementary_data.zip › FigureS3.jpg]

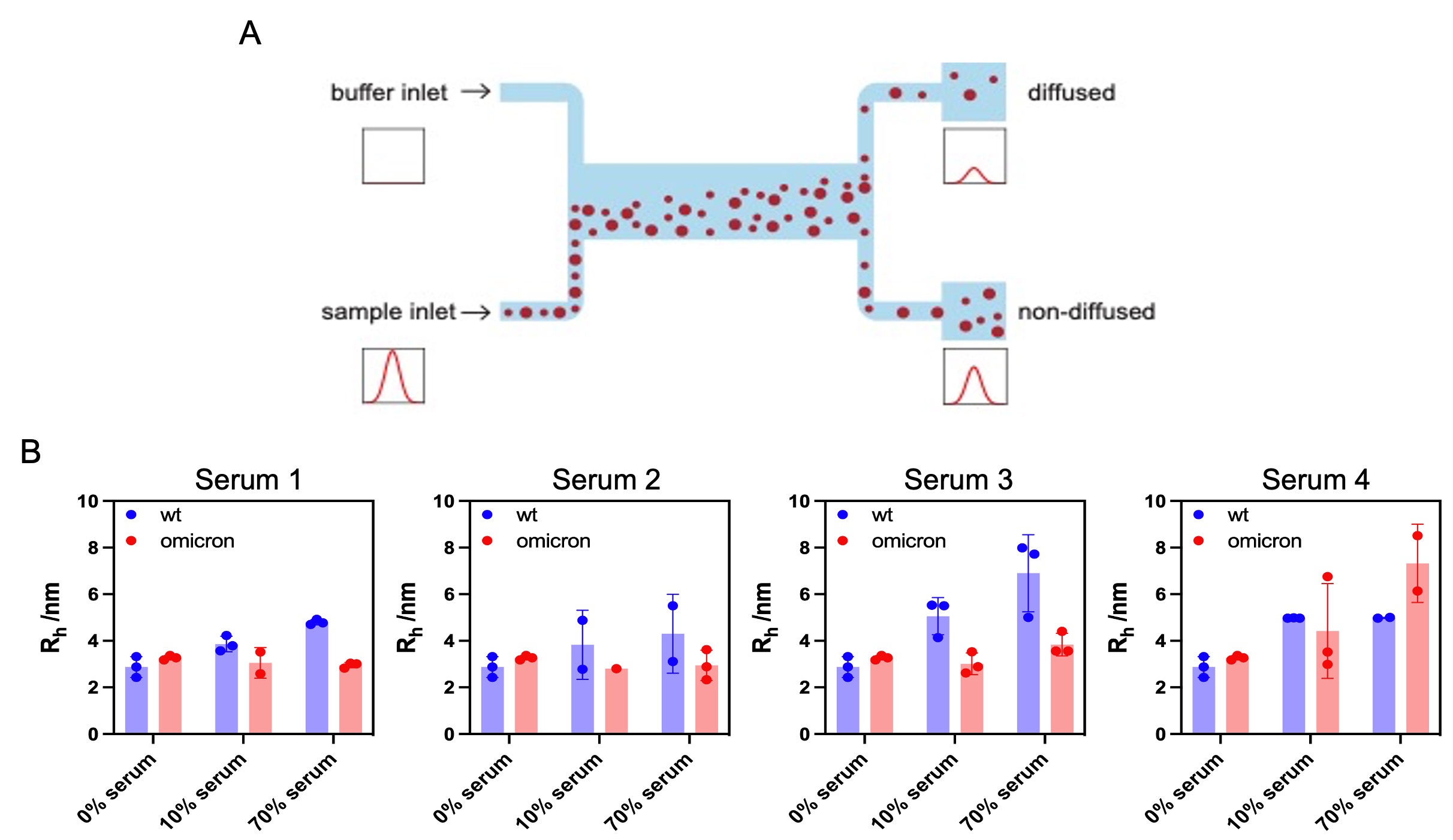

Supplement: jiad135_Supplementary_Data [file jiad135_supplementary_data.zip › FigureS4.jpg]
